# An Overview on Leishmaniasis in Romania: Diagnosis and Therapeutics

**DOI:** 10.3390/tropicalmed7110334

**Published:** 2022-10-28

**Authors:** Florentina Daraban Bocaneti, Larisa Maria Ivanescu, Liviu Miron, Oana Irina Tanase, Mihaela Anca Dascalu

**Affiliations:** 1 Department of Public Health, Faculty of Veterinary Medicine, Iasi University of Life Sciences Ion Ionescu de la Brad, 700489 Iasi, Romania; 2 Department of Clinics, Faculty of Veterinary Medicine, Iasi University of Life Sciences Ion Ionescu de la Brad, 700489 Iasi, Romania

**Keywords:** leishmaniasis, Romania, epidemiology

## Abstract

Leishmaniasis, a vector-borne disease considered to be one of the twenty neglected diseases by the World Health Organization, represents one of the public health concerns in endemic countries. In humans, as well as in animal counterparts, the infection can evolve with different clinical localizations, such as those that are cutaneous, mucocutaneous and visceral. Romania has been traditionally considered a nonendemic country for *Leishmania* species infection and has had sporadic positive human cases; however, the climate change recorded in recent decades has created potentially optimal conditions for the preponderant vectors of *Phlebotomus* spp., which has lately been identified in various parts of country. Moreover, with people and dogs (the prevailing hosts) traveling in endemic countries, the disease was imported and diagnosed in both species, and became a medical concern. In this review, we focused on the: (1) epidemiological data of leishmaniasis cases, both in humans and animals, reported by Romania; (2) diagnostic tools available for confirmation since there is a lack of gold-standard laboratory methods for human and dog patients; and (3) conventional antileishmanial therapy.

## 1. Introduction

Leishmaniasis, a vector-borne disease considered to be one of the twenty neglected diseases by the World Health Organization (WHO), represents one of the public health concerns in endemic countries [1]. Leishmaniasis is induced by parasites belonging to the genus *Leishmania* (*Leishmania infantum*, *Leishmania major*, *Leishmania donovani*, *Leishmania tropica* and *Leishmania chagasi*) that are able to multiply in different vertebrates, which represent the infection’s main reservoirs [2].

Particularly, most transmission cycles are zoonotic, with many species of rodents, wild and domestic canids, and a variety of other mammals serving as reservoir hosts and phlebotomine sand flies acting as vectors [3]. Human beings are infected by *Leishmania* species through the bite of vectors which have initially fed on an infected host. Interestingly, the infection’s clinical evolution depends on the species of *Leishmania* involved and on the immune system’s capability to overcome that infection [4].

The *Leishmania* life-cycle involves two well-known stages: the first stage, or the promastigote insect stage, and the second stage, or the amastigote vertebrate stage. In the first stage, promastigotes replicate and differentiate in the gut of vectors (hematophagous female sand flies) [5]. Further, when the vectors are feeding, metacyclic promastigotes will be inoculated into the mammalian host’s dermis. In the host, *Leishmania* has an affinity for immune cells, infecting phagocytes, such as neutrophils, dendritic cells and, particularly, macrophages. Thus, the promastigotes develop into amastigotes, which multiply inside parasitophorous vacuoles in phagocytes [3,5,6].

From the clinical point of view, leishmaniasis is differentiated into four major syndromes: cutaneous leishmaniasis (CL), mucocutaneous leishmaniasis, visceral leishmaniasis (VL) and post-kala-azar dermal leishmaniasis. Each syndrome differs in the degree of severity, although VL is considered potentially fatal with the highest mortality rate reported, especially when adequate, accessible treatment options are lacking [4]. VL occurs in more than 65 different countries; the vast majority of positive cases are reported in Asia (Bangladesh, India and Nepal), Africa (Sudan) and south America (Brazil) [6]. VL is known to result following the infection of phagocytes within the reticuloendothelial system due to metastasis of parasites and parasite-infected macrophages from the initial site of cutaneous infection. Moreover, because of parasites’ affinity for macrophage cells found in the liver, spleen and bone marrow, a progressive hepatosplenomegaly and bone marrow suppression may result and, unless treated, patients develop pancytopenia and immunosuppression [2]. Thus, in persons with human immunodeficiency virus/acquired immunodeficiency syndrome (HIV/AIDS) or other causes of cell-mediated immunosuppression, VL is seen as an opportunistic infection [4].

Cutaneous leishmaniasis (CL) is considered the most common leishmanial syndrome and is reported to be endemic in more than 70 countries worldwide [7]. This syndrome is characterized usually by painless and chronic skin lesions, often occurring at sites of infected vector bites, on uncovered areas of the body such as the face, forearms and lower legs. Uncomplicated cutaneous lesions are often self-healing but, in some situations, they may progress with mucocutaneous tissue involvement. Additionally, in diffuse cutaneous disease, nodular lesions of different sizes erupt at various locations often that are distant from the site of insect exposure [2,7]. Annually, it is estimated that there are around 600,000–1,000,000 new cases globally, and a high proportion is reported by Syria, Brazil, Colombia, Iraq, Peru and Sudan [5,7].

Mucocutaneous leishmaniasis can be markedly disfiguring and even life-threatening; it usually occurs after the apparent resolution of CL, although it can simultaneously exist with skin involvement. The route of infection spread may be either hematogenous or lymphatic, whilst the lesions normally appear within 24 months of cutaneous infection [2]. Surprisingly, the underlying mucocutaneous leishmaniasis pathogenesis is not well understood and is probably a consequence of a complex interplay of host and *Leishmania* species factors. The nasal and oral cavities and eyelids are mostly affected; often, ulcerative lesions may extend into the oropharynx and the trachea, which in turn, may impair the respiratory function [2,6].

Post-kala-azar dermal leishmaniasis is considered the main complication of VL, characterized by different cutaneous lesions that primarily develop around the oral cavity and from where it spreads to other parts of the body [8]. The pathogenesis of post-kala-azar dermal leishmaniasis (PKDL) is not fully understood, but appears to be related to an aggressive interferon γ-driven host immune response generated against parasites. Moreover, as a consequence of a high prevalence of PKDL after VL treatment in Sudan and India, it has been suggested that inadequate treatment regimens (mostly with stibogluconate and liposomal amphotericin B) may be an important event for developing this syndrome [2,8].

A precise identification of the *Leishmania* species has a major clinical impact, since it will direct the selection of an adequate treatment [9]. In practice, failures in leishmaniasis treatment efficiency have been often reported lately, which are due to chemoresistance of the parasite [10].

Therefore, in this review we focused on the epidemiological data of leishmaniasis cases, both in humans and animals, reported by Romania; on the diagnostic tools available for confirmation since there is a lack of a gold-standard laboratory methods, both for human and dog patients; and on conventional antileishmanial therapy.

## 2. Epidemiological Data of Leishmaniasis in Humans and Animals Reported in Romania

### 2.1. Autochthonous and Imported Animal Cases

Since the first case (with no history of traveling to endemic areas or direct contact with a positive case) of autochthonous canine leishmaniasis (CanL) reported in 1934 by Mihailescu et al., a new case was confirmed by Mircean et al. in 2014 in a dog with no history of traveling or living abroad; this came after more than 80 years since the last positive case. Several determinations, such as a complete blood count, serum biochemistry, and histological and microscopical examinations, along with the qualitative detection of *L. infantum* immunoglobulin G (IgG) by a rapid diagnostic test, confirmed the CanL [11,12].

Only a few imported CanL cases have been reported throughout Romania. Consecutively, in 2014, a case of imported CanL from Spain was confirmed in Iasi County by Pavel et al. (2017) [13]. The dog was born and lived in Spain for three years; six months after its arrival in Iasi County, the first signs occurred. Diagnosis was confirmed by performing a complete blood cell count, serum biochemistry, and fine-needle aspirates from prescapular and popliteal lymph nodes, followed by a May–Grunwald–Giemsa staining that showed numerous amastigotes; the deoxyribonucleic acid (DNA) extraction, nested polymerase chain reaction (PCR) and the subsequent sequencing confirmed the *L. infantum* species with 100% homology based on the SSUr-RNA sequence. Because the dog’s condition worsened, the owner decided on euthanasia.

Three years later in 2018, Tanase et al. reported another imported case of CanL, also in Iasi County, located in northeastern Romania. The dog was born and lived in Italy, but the owners confirmed that they traveled often with the dog to Romania. The initial clinical signs noted by the owner occurred 30 days upon their arrival in Romania. Diagnosis was confirmed via an ELISA. However, the light microscopy of stained smears did not reveal the parasite. Serum biochemistry, a complete blood cell count and radiography were used in the diagnosis protocol. The treatment of the dog consisted of the administration of N-methylglucamine antimoniate (Glucantime) for two months, whereas allopurinol was recommended for eight months, with an improved general body condition after treatment [14].

In 2018, the latest case of imported CanL was confirmed by Toma et al. in Cluj-Napoca, Romania. The dog was born and lived in Florence, Italy, and was moved to Romania. The diagnosis of CanL was confirmed by using a series of tests, such as: a complete blood cell count, serum biochemistry, cytology of lymph node aspirates and of the internal part of the crusts, qualitative immunoassay, and real-time PCR. Taking into consideration the severity of the clinical signs, along with the zoonotic concern, it was recommended that the dog be euthanized [15].

### 2.2. Animal Screening Studies

Only a few studies were published in Romania concerning the prevalence of CanL. In this regard, in 2012 Hamel et al. performed a screening using molecular, serological and hematological methods for canine arthropod-borne pathogens, including *Leishmania* spp., on 138 samples collected between 2009 and 2010 from Bucharest, Romania. The indirect immunofluorescence antibody test (IFAT) revealed four positive cases of *Leishmania* spp., from which three dogs were presented to a private clinic from Bucharest and one dog was classified as imported [16].

A few years later, in dogs from Ramnicu Valcea, Romania, the presence of CanL was demonstrated by using PCR and serology [17]. The study was performed on serum, blood and conjunctival swabs collected in 2014 from 80 dogs. A real-time quantitative PCR (RT-qPCR) was applied on blood and conjunctival samples, and seven dogs were found to be positive by conjunctival swabs and one dog by blood sample. The sera tested for anti-*Leishmania* antibodies via an enzyme-linked immunosorbent assay (ELISA) revealed three seropositive dogs, whilst four showed borderline results, which highlight the exposure or infection.

Although wildlife is considered to have a minor reservoir role for CanL [18], an extensive study was conducted by Ionica et al. (2017) on 514 red foxes’ conjunctival secretion swabs collected between 2016 and 2017, coming from ten counties throughout Romania. After DNA extraction, a real-time PCR (RT-PCR) amplification and TaqMan minor groove binder (TaqMan-MGB) assay were performed, and all samples proved to be negative for *L. infantum* DNA. However, taking into consideration the increased number of leishmaniasis cases throughout Europe and the actual national epidemiological situation, the contribution of red foxes should not be disregarded in the future [19].

A study focused on the detection of an extended variety of vector-borne pathogens in wildlife [20] reported that out of 54 Eurasian golden jackals legally hunted between October 2013 and May 2015 in 11 counties of Romania, only 1 animal proved to be PCR-positive for *L. infantum*, which was also confirmed by sequencing of ITS1-5.8S. Notwithstanding the low prevalence obtained, the presence of *L. infantum* in this species deserves closer attention, as territorial movements of jackals could represent a major risk for CanL spreading into new areas and also may represent reservoirs for various carnivores’ vector-borne diseases. A more recent study was published by Cimpan et al. (2019) [21], targeting two Romanian counties for CanL prevalence. In 2017, 110 dog’s sera were collected from Galati and Calarasi Counties and tested by using an ELISA for anti-*Leishmania* spp. IgG. Positive samples (5/60) were obtained only from Galati County, with a total seroprevalence of 8.33%. The latest study published in 2020 by Cazan and coworkers aimed to analyze the prevalence of various vector-borne infections caused by different pathogens, including *Leishmania infantum* in a dog kennel from Arges County, known to have a history of positive cases. In this respect, 149 blood and conjunctival swab samples were collected in 2017 and tested further for the presence of anti-*L. infantum* antibodies using a rapid test and a commercial kit, but with no positive results. Next, genomic DNA was extracted and RT-qPCR and TaqMan-MGB assays were performed; surprisingly, 30 positive samples for *L. infantum* DNA were revealed [22].

### 2.3. Human Cases of Leishmaniasis

Human leishmaniasis usually evolves with different clinical forms, where the visceral form is considered to be the most dangerous and the evolution is potentially lethal without treatment [23].

In Romania, the first case of autochthonous VL was reported by Manicatide in 1912, followed by 24 other cases confirmed in 1954 as part of an infantile focus in the southern part of country [24], and followed by 2 other cases in Prahova and Giurgiu Counties. A few years later, between 1999 and 2004, the first two Romanian imported cases of VL were reported in humans with a history of living in rural regions of Greece and Spain in close contact with stray dogs. The cases were diagnosed at the Clinical Hospital of Infectious and Tropical Diseases in Bucharest [25]. After a short period, a new report confirmed another three patients with VL imported from Spain, which were diagnosed at the Victor Babes Hospital in Timisoara, in 2005 [26]. For the first patient, diagnosis of VL was confirmed by bone marrow biopsy, which revealed *Leishmania* bodies in the Giemsa-stained films. The main clinical signs observed consisted of fever, headache, general discomfort, weight loss, tachycardia, cutaneous pallor and facial hyperpigmentation; additionally, hepatomegaly and splenomegaly were also found. Although the specific therapy with liposomal amphotericin B (3 mg/kg/day) was administered, the patient died following coma and cardiorespiratory failure. For the second patient, the clinical signs consisted of fever, headaches, asthenia and general discomfort, accompanied by hepatomegaly and splenomegaly; in this case, the diagnosis of VL was established also by bone marrow biopsy. The same specific therapy as mentioned above was administrated for five days and the evolution was good compared to patient 1. For the third patient, diagnosis and therapy of VL underwent the same protocol as for the first two patients; the following clinical signs were reported: fever, diffuse abdominal pain, purplish-red leg nodules, polyarthralgia, joint stiffness and necrotizing vasculitis of the fifth left toe, simultaneously with comorbidities; no follow up was available for this patient [26].

Gaman et al., 2010 reported another case of VL in a patient from Sopot, Dolj County who traveled to Greece for several months. The infection was confirmed by an immunoelectrophoretic test and by bone marrow biopsy. The clinical signs presented by the patient were represented by an irregular fever, fatigue, weight loss, pallor of the skin, left bacterial otitis and transient period of obnubilation, along with splenomegaly, hepatomegaly and enlarged lymph nodes. Unfortunately, the patient presented some associated comorbidities (hepatitis B and C infections), which led to an unfavorable prognosis. The patient was symptomatically treated and redirected to the Infectious Diseases Clinic [23]. Furthermore, a few years later, two cases of VL with the *Leishmania donovani* complex were confirmed at the Fundeni Clinical Institute, Romania [27]. The first patient, originally from Dolj County (with a history of work in Italy for three years), presented with weight loss and abdominal hypochondrium pain, especially during effort. The diagnosis was confirmed by blood analysis, abdominal ultrasonography, bone marrow and hepatic biopsy. In this case, two possible origins of infection were considered: the endemic area of Italy or the Oltenia region (as an autochthonous source), where the patient was living. For the second patient, originating from Iasi County, the infection origin could not be established; however, the possibility of an autochthonous infection was considered since the patient worked in the southwest of Romania, where previous leishmaniasis cases were reported. The patient presented with fever, tachycardia, polypnea, icterus and splenomegaly. Following the liver biopsy, the amastigotes were revealed in histiocytes, which confirmed the diagnosis [27]. A more recent case of imported VL was reported in 2014 by Alexa et al. The case was confirmed in a 44-year-old female that had worked for six months in Italy. She presented to the hospital with dehydration, pallor of the skin, normothermia, asthenia, vertigo, perspiration, weight loss and menometrorrhagia (as she had a history of uterine fibroma), and splenomegaly. Diagnosis of leishmaniasis was confirmed by bone marrow aspiration. The patient received a treatment with liposomal amphotericin B, but unfortunately, given the comorbidities, the patient had an unfavorable outcome [28].

## 3. Diagnostic Tools Available for Leishmaniasis Confirmation

Leishmaniasis represents a complex disease with various clinical manifestations, whereas its ethology consists of different species belonging to the genus *Leishmania*. Nowadays, due to considerable advances in diagnostic techniques, a high number of confirmatory tests are available; however, many of these tests have limitations, since they do cross-react with other parasites and their sensitivity rate is correlated with the clinical outcome. In leishmaniasis endemic areas, there is a high rate of asymptomatic infected people, which is a well-recognized problem reported for years, making the diagnosis difficult [29].

### 3.1. Clinical Diagnosis

The first clinical sign related to *Leishmania* spp. infection is a reduced rash at the bite site. Secondary to rash formation, the parasites’ multiplication causes an inflammatory reaction, whereby the rash develops into an open cutaneous ulcer or is disseminated to organs such as the spleen and liver. These inflammatory responses depend on the parasite species, on its host, on immunity status or on the strain, without being able to provide a diagnosis of certainty [5,30].

### 3.2. Conventional Diagnosis

The conventional diagnosis consists of microscopic identification of amastigotes in aspirates from various organs such as bone marrow, lymph nodes, liver, skin and spleen, or by parasite culturing; both remain essential techniques in epidemiological studies [31,32]. The diagnostic sensitivity of smears prepared from peripheral blood has been low, especially in patients with low parasitemia [33], and is higher in HIV-infected individuals [30]. Splenic aspirates offer the highest sensitivity (93.1–98.7%) [32], but the risk of sampling is also higher and can result in fatal hemorrhages [32,33]. These techniques are quite invasive and uncomfortable for patients, while cultivation on culture media is expensive, difficult to perform and time-consuming [5]; however, the isolation and culture of parasites offer a higher diagnostic sensitivity. Combining the culture technique with the multilocus enzyme electrophoresis technique, it is possible to obtain a more accurate characterization and identification [30]. Different *Leishmania* spp. strains have been maintained as promastigotes in artificial culture media. For the conversion of amastigotes to promastigotes, biphasic culture media might be used, whereas a monophasic medium is preferred for the amplification of parasite numbers [34,35].

In Romania, the diagnosis of leishmaniasis is mostly performed via bone marrow biopsy, followed by a Giemsa-stained blood smear analysis; moreover, this procedure is considered the standard diagnostic method. On the contrary, serology and parasite DNA amplification are not commonly used because the Romanian national insurance system does not cover the high costs of these specific laboratory tests [36].

The sensitivity of parasitological examination is considerably lower when the samples are collected from lesions with rare amastigotes, a feature that influences the diagnosis of mucosal leishmaniasis [37]. Given these premises, for a mucosal leishmaniasis diagnosis the parasitological examination is not recommended; therefore, the confirmation is based on a PCR, Montenegro’s intradermal test (MDI), histopathological examination or in vitro isolation of parasites [37]. Issues related to laboratory infrastructure and technical skills should be considered in order to choose the right method (Table 1).

### 3.3. Serological Diagnosis

For the diagnosis of VL, various serological tests have been described; the most recommended are the ELISA, IC and IFA. Besides the aforementioned assays, other laboratory tests may be performed, e.g., the direct agglutination test (DAT), the fast agglutination screening test (FAST), the complement fixation test (CFT) or the Western blotting test (WB). The high range of sensitivity and specificity depends mainly on the protocol and on the case symptomatology. Over time and based on specific humoral responses, other serological tests have been developed. For instance, the immunodiagnosis test was created to identify the antigen in tissue, blood or urine samples, to detect nonspecific or specific antileishmanial antibodies (immunoglobulin), and to evaluate the leishmaniasis-specific cell-mediated immunity [33]. Accordingly, these serological methods have been classified as nonspecific and specific [34].

### 3.4. Nonspecific Tests

The formalin gel test, the aldehyde test and Chopra’s antimony test are classified as nonspecific methods and were mostly used in the past. Their efficiency depends on globulin concentration; thus, the positive results may be influenced by a variety of conditions. Since these nonspecific tests showed a low specificity and a variable sensitivity, they have been replaced by more accurate or specific tests [49].

### 3.5. Specific Tests

The tests based on antigen detection proved to be more accurate than antibody-based immunodiagnostic tests [34]. Moreover, such tests showed their efficacy in confirming the human cases with deficient antibody production. Surprisingly, De Colmenares et al. (1995) reported that kala-azar patients shed, through urine, two polypeptide fractions of 72–75 kDa and 123 kDa [50], and the identification of these fractions was associated with a promising sensitivity and specificity. Moreover, in some preliminary trials, a new latex agglutination test aiming to detect the leishmanial antigen in the urine of patients with VL showed similar results [39].

According to the international standards described by Herrera et al. (2019), the internal indirect immunofluorescence test using the crude *Leishmania infantum* MHOM/COL/CL044B promastigote antigen proved to be irrelevant due to a cross-reactivity with *Trypanosoma cruzi* epimastigotes [45]. The use of LAg (*Leishmania antigen)* in the diagnosis of human VL caused by *L. donovani* or *L. infantum* has been validated in different diagnosis centers from a few countries, such as India, Nepal, Brazil, Ethiopia and Spain [43]. However, this technique has some limitations, such as the probability of a cross-reaction with other trypanosomatids or the low sensitivity in detecting asymptomatic cases [51].

### 3.6. Enzyme-Linked Immunosorbent Assay (ELISA)

This assay represents one of the most sensitive serodiagnosis methods for VL, although its sensitivity depends on the antigen used [52]. The crude soluble antigen extracted from live promastigotes is able to detect IgG and IgM in patients with visceral and cutaneous leishmaniasis. On the other hand, in asymptomatic patients or in those in the early stage of infection with low specific antibody concentrations, a high number of false negatives have been reported [38]. Thus, in order to increase sensitivity, different antigens have been used, such as the immunodominant K39 protein specific to *L. donovani* [53], as well as combinations with different recombinant antigen subunits such as rK9, rK26 and rK39, or Lb8E and Lb6H derived from *Leishmania braziliensis* A [52,54].

### 3.7. Immunochromatographic Tests

Various immunochromatographic tests were demonstrated to be important tools in VL diagnosis, mainly due to their simplicity, low costs and expeditive results. Although the vast majority of these rapid tests have shown good performances, no definitive diagnosis should be established exclusively based on their results; therefore, before implementing any therapy, a clinical and epidemiological corroboration would be necessary. Indeed, in order to reduce false positive or false negative results, the diagnosis confirmation, performed by using microscopy, is mandatory [44,45,51,55].

### 3.8. PCR Diagnosis

Several sequences of the *Leishmania* genome, such the genes for ribosomal RNA, β-tubulin, gp63 locus, HSP70 locus, cysteine proteinases or kinetoplast DNA (kDNA) minicircles, have already been described as targets for amplification. According to different studies based on PCR diagnosis, it was observed that the ideal target region was the kDNA-PCR, followed by the SSU rRNA-PCR, ITS-PCR, ITS2-PCR, ITS1-PCR, ME-PCR and HSP70-PCR [40]. Moreover, for leishmaniasis diagnosis, the kDNA-PCR proved to be sensitive and may be used as a standard method for routine diagnosis when species identification is not required. Different studies on human CL and VL cases reported a wide range of sensitivity for the ITS1-PCR, ME-PCR and HSP70-PCR [40,56]. In patients with mucosal leishmaniasis, a high sensitivity has been noted when the amplification targeted the blood kDNA and HSP70-PCR [56]. Moreover, in cutaneous and mucosal leishmaniasis confirmation, a real-time PCR (qPCR) protocol targeting the HSP70 gene showed a higher sensitivity compared to the direct parasitological examination via microscopy [56,57].

### 3.9. Real-Time PCR

In order to differentiate the subgenera *Leishmania* and *Viannia*, and to distinguish *L. infantum* from *L. amazonensis,* an approach based on two qPCRs (qPCR-ML and qPCR-ama) targeting the minicircle kDNA followed by a melting analysis has been developed [42]. Thus, based on the promising results, the RT-PCR technique was continuously developed and improved, leading to results indicating that the Linj31-qPCR was able to identify parasites belonging to the subgenus *Leishmania* without cross-amplification to another parasite subgenus [41]. Moreover, the qPCR technique is recommended for the quantification of the parasite load, but also for monitoring the cure in patients [42].

### 3.10. Nested and Semi-Nested PCR

This type of PCR makes a crucial contribution to differentiating the species [58]. For amplification, two different primer pairs are used in two consecutive cycles. The second primer pair is able to amplify the secondary target found on the first amplification product. Although this method is highly sensitive, it yet has some limitations, mainly because of the multiple pipetting steps and transfers, which, in turn, increases the chances of contamination [5].

### 3.11. MALDI-TOF Mass Spectrometry

Within the last decade, the identification of intact bacteria by mass spectrometry was a significant step in microbiology. Now, more and more clinical laboratories use matrix-assisted laser desorption time-of-flight for rapid microorganism identification, including *Leishmania* spp. [59].

With respect to veterinary medicine, various immunological tests have been extensively used in order to confirm dog leishmaniasis, most likely of the visceral form. However, because of specific immune complexes, false negative results are often reported [60]; therefore, the sensitivity and specificity are strongly correlated with the antigen type used during immunological testing. It is noteworthy to mention that in Brazil, special diagnosis protocols have been proposed in order to overcome this limitation. Thus, the first step in dog leishmaniasis confirmation consists of the previous testing based on the rK28 antigen, followed by an ELISA based on the crude antigen [47]. In a symptomatic dog with VL, a high sensitivity was recorded via an agglutination test prepared with the crude *L. infantum* antigen, whilst rKE16 and rA2 antigens developed a lower sensitivity [61]. Relying on these reports, in the early diagnosis of VL, only the promastigote antigen is recommended [62].

The immunofluorescence assay directed at antibody detection, as well as the ELISA and Western blotting tests, have been effective in assessing the infection status, allowing an early leishmaniasis confirmation. Different antigens, such as chaperonin HSP60, 32 and 30 kDa (kilodalton) antigens, may be of importance in establishing an early diagnosis of leishmaniasis, as demonstrated by the identification of these antigens in dogs under experimental infections. Reactivity to HSP83 and HSP70 seems to occur mostly in advanced infection. In a study conducted in Brazil, it was reported that the use of the crude antigen in a chemiluminescent ELISA demonstrated a sensitivity of 75% and a specificity of 73.3%, whereas the use of the multi-epitope protein, PQ10, showed a sensitivity of 93% and a specificity of 80% [46,48].

### 3.12. Clinical Diagnosis in Veterinary Medicine

Studies conducted on batches of dogs infected with *Leishmania* spp. showed that the most common clinical sign was lymph node enlargement, noted mostly 12 weeks postinfection. The most common systemic sign observed in up to 50% of infected dogs was represented by progressive weight loss, occurring 18 weeks postinfection. Cutaneous signs were usually developed 18 weeks postinfection, whereas the ocular signs were very rare and occurred very late, after 22 weeks postinfection. The most significative lesions appeared one-year postinfection; the earliest change was thrombocytopenia and/or mild nonregenerative anemia. Both thrombocytopenia and/or mild nonregenerative anemia were reported to progressively increase in frequency, and eventually, may have affected between 71 to 87% of the infected dogs that survived up to 22 weeks. Reversal of the albumin/globulin ratio was recorded in late infections, but was not always associated with total protein augmentation. However, various studies have reported that in actively infected dogs, an asymptomatic status is only temporary and progression to clinical disease appears to be mandatory [46,63,64].

## 4. Conventional Antileishmanial Therapy

The conventional cure of leishmaniasis is based on systemic drugs such as antimonials, amphotericin B, nucleoside analogues, paromomycin and miltefosine. Despite the actual progress in medicine, no efficient vaccine has been formulated in order to prevent leishmaniasis. In this section, several drugs accessible for antileishmanial chemotherapy will be detailed.

### 4.1. Pentavalent Antimonials

Pentavalent antimonials have been administrated for more than six decades as the first-choice treatment for the major leishmaniasis syndromes in most parts of the world. At the beginning of the XX century, the efficacy of antimony potassium tartrate in curing mucocutaneous leishmaniasis was reported by Gaspar Vianna, who is considered to be the initiator of the antileishmanial treatment [65]. The studies from the beginning proposed that sodium stibogluconate presents certain mechanisms of action, such as impairing macromolecular biosynthesis in amastigotes, possibly through inhibition of *Leishmania*’s various energy-generating metabolic processes, including ADP phosphorylation, glycolysis or fatty acid oxidation [66,67]. More current studies have suggested that apoptosis is an important event in Sb(III)-treated amastigotes [68]. Antimonials are reduced through different mechanisms, including enzymatic and nonenzymatic reactions [69,70]. It was demonstrated that the transformation of a pentavalent antimonial to a trivalent antimonial through a nonenzymatic reaction is higher at a reduced pH, which imitates the intracellular amastigote form as compared to promastigotes found extracellularly [71].

However, since 1990, the resistance against pentavalent antimonials has emerged rapidly in India and Sudan due to widespread misuse of the drug (low dose and duration, interruption of treatment), the unrestricted use by qualified and unqualified medical personnel, and ground water contamination with arsenic [72]. Due to its effectiveness and reduced cost, it is still used as the gold standard in some old world and new world countries [10].

Available products on the market include sodium stibogluconate (Pentostam) and meglumine antimoniate (Glucantime). They have a reduced capacity for oral absorption, and therefore, intramuscular or intravenous administration is preferred, being rapidly absorbed. In patients with VL, a regimen of 20 mg/kg for 20 to 30 days is recommended; however, the main disadvantage is represented by agent accumulation in internal organs as well as some adverse effects (hepatitis, nausea, myalgia and pancreatitis) [73,74,75]. In many countries, antimonials are considered first-line agents for the treatment of leishmaniasis, but the present scenario in Romania is different since, in recent decades, it was not considered an endemic area. Therefore, for none of the human positive cases reported in recent decades were the pentavalent antimonials administrated [26].

### 4.2. Amphotericin B Deoxycholate/Liposomal Amphotericin B

In recent decades, an unwanted resistance against antimonials has been reported more and more. Most of the therapeutic alternatives have the inconvenience of being limited in certain leishmaniasis patients, such as pediatric and HIV patients.

Amphotericin B deoxycholate (Amp B-Doc) is known as an antifungal molecule generated by *Streptomyces nodosus* (filamentous bacterium) [76]. Taking into consideration the toxicity, costs and difficulties of intravenous administration in endemic areas, a diffuse administration of the drug has been impossible [77]. This drug demonstrates selective activity against *Leishmania* and *Trypanosoma cruzi*. Ergosterol, the main sterol in these parasites, is responsible for this selectivity, for which amphotericin B has a higher affinity over cholesterol, the principal sterol in the mammalian infected cells [78]. The main action of this product consists of binding to the *Leishmania* cell membrane, which in turn, leads to the organization of micropores that causes a high cell permeability and lysis [79]. Other, different pathways are also described, such as the formation of reactive oxygen species (ROS) and auto-oxidation, which were also related to cellular lysis [79,80].

In practice, Amp B is used as a suspension given mainly via the intravenous route (IV) because of its low oral absorbance capacity; once administrated, the drug is dissociated mainly by the hepatic tissue, and subsequently eliminated through urine and gall bladder secretion. However, an Amp B regimen is accompanied by important side effects related to a decreased level of K+ ions, including renal failure, or worse, anaphylactic shock [81].

In order to counteract all the limitations of Amp B administration, new lipid formulations were discovered, such as the liposome amphotericin B (AmBisome) that has a lower toxicity. Interestingly, the new formula possesses a particular biomechanism consisting of a predilection for hepatic and splenic reticuloendothelial cells, which in turn, results in an improved therapeutic concentration that is highly desired in particular patients, such as those with immunosuppression or visceral forms [82]. In order to compare the efficiency of the liposomal formulation versus the classical Amp B, several studies have been conducted, demonstrating a better excretion capacity and an increased plasma concentration of the liposomal form when compared to Amp B-Doc, although no important differences regarding the half-life were observed [83]. The intravenous dosage regimen differs between the endemic regions, such as 30–50 mg/kg, 24–35 mg/kg and 18–21 mg/kg, whereas a dose of 3.75 mg/kg seems to be an efficient cure in more than 85% of patients with the visceral form from India [84]. However, in the last decade, resistance to Amp B in the clinical isolates has been reported, and consecutively, several mechanisms were incriminated, such as an alteration in the membrane structure, increased mRNA levels of the MDR1 gene and the overexpression of trypanothione biosynthesis pathway proteins [85].

The WHO has approved liposomal amphotericin B as an efficient cure of VL, which was subsequently authorized in the United States of America as well as in a few European countries, particularly Romania. In the case of Romanian patients with confirmed VL, a therapy with liposomal amphotericin B was initiated, with a dose of 3 mg/kg/day for five to six days. In nonendemic countries (e.g., Romania), given the clinical outcome of VL which may resemble other pathologies, the differential diagnosis sometimes is laborious and leads to delayed diagnosis, a situation that is familiar for the Romanian physicians [24,27].

### 4.3. Miltefosine

Miltefosine is considered to be the first agent compatible with the leishmaniasis oral therapy and is an alkylphosphocholine drug. Since 2010, it has been included by the WHO on the List of Essential Drugs, and is considered, at the moment, to be the only oral formula active in the cure of *Leishmania* infection [86]. Preliminary antileishmanial activity for miltefosine was noted in animal studies [87]. Miltefosine acts by killing the parasite both in vivo and in vitro through different mechanisms, such as by impairing the intracellular signaling pathways, resulting in apoptosis. Interestingly, other several apoptotic mechanisms have been well described, including modifications in cellular membrane compositions by decreasing the phospholipid metabolism and alkyl lipid metabolism, or by increasing the expression of various enzymes in host macrophages, such nitric oxide synthetase 2, which is responsible for the synthesis of a compound harmful for *Leishmania* survival [88].

In endemic regions, such as Southern Asia and Eastern Africa, the administration of miltefosine demonstrated a high efficiency, whereas in patients with VL, regimens of 2.5 mg/kg/day for 28 days were recommended. Unfortunately, after about ten years of administration, an increased resistance has been reported in some endemic regions, mainly because of its improper use and long shelf life [89,90]). Taken into consideration all these factors, at the moment, miltefosine is not recommended as a monotherapy; therefore, a combination therapy with other drugs has been initiated [91]. Currently, miltefosine is authorized in several countries, but not in Romania. The ample use of miltefosine raises some major concerns related to its teratogenic potential, liver side effects and its long half-life [89].

### 4.4. Nucleoside Analogues

At the beginning of the 1980s, a new agent (allopurinol) was started to be considered as a potential cure of VL and CL. Its efficiency has been tested alone and in combination with antimonials in different clinical trials, and was concluded to be considered an ineffective product for the treatment of human leishmaniasis [92].

Several bioactivities are well understood, including the capacity of inhibiting some certain enzymes involved in the salvage pathway of purine synthesis or the transformation of nucleoside triphosphate analogues [93], all known to impair molecular biosynthesis [94]. Clinical pharmacology studies on oral allopurinol administration showed that, in contrast to plasma concentration in dogs, plasma levels in humans were decreased, with a reduced absorption and transformation by enteric gut flora [95]. Despite its failure in curing human leishmaniasis, in veterinary medicine, allopurinol has gained extensive use in the treatment of canine leishmaniasis [92].

### 4.5. Paromomycin

First isolated in 1956, paromomycin (Aminosidine) is a broad-spectrum aminoglycosidic aminocyclitol produced by *Streptomyces riomosus var. paromomycinus.* Initially, paromomycin was advised for treatment in intestinal infections with the protozoal organism *Entamoeba histolytica*, whereas later, its administrations were extended to a broad spectrum of infections, including VL [96].

The main bioactivity mechanism consists of protein synthesis inhibition simultaneously with membrane permeability changes. Furthermore, it was demonstrated that after 72 h of contact between paromomycin and *L. donovani*, the functionality of mitochondria was affected, suggesting a possible drug activity through a mitochondrial-mediated pathway [96].

In the first instance, the Food and Drug Administration (FDA) authorized paromomycin mainly for *Entamoeba histolytica* infection treatment; after oral administration, the drug has a low rate of digestive assimilation, resulting in a high proportion of the unchanged dose being eliminated through the feces. Instead, following parenteral injections, the elimination mainly occurs through the urine, whereas an important collection occurs in kidney and inner-ear components [10]. However, post-treatment side-effects have been reported, including pain at the site of infection, hepatotoxicity, renal toxicity and ototoxicity [85]. The development of paromomycin resistance is the main consequence of administrating paromomycin as monotherapy; therefore, for a successful VL therapy, a polytherapy is advisable [97]. In Europe and Romania as well, the current form of paromomycin in veterinary medical marketing is the paromomycin sulfate, used in a concentration of 175 mg/mL solution for injection.

## 5. Conclusions

Leishmaniasis is considered to be a neglected tropical disease and is registered as a global problem by the WHO. Since Romania is annually registering a relatively low number of cases, both in human and animals, doctors are unfamiliar with the clinical symptoms; therefore, many times in cases patients may by misdiagnosed and the therapy is given late, leading to fatal cases. However, recent diagnostic procedures such as advanced molecular biology techniques, together with specific and efficient drug therapy accessibility, seem to be revolutionary in the prognosis of fatal cases of leishmaniasis.

## Figures and Tables

**Table 1:** Various assays for leishmaniasis diagnosis confirmation.

Assay	Sensitivity	Specificity	Comments	References
Parasitological diagnosis: light microscopy	From 93.1 to 98.7% in splenic aspirate, or 52% or less in lymph node aspirate and peripheral blood smear.	100%	For the culture, sophisticated laboratories are required and techniques can be risky for the patient.	[30,31,32,38]
Antigen detection: latex agglutination test (KATEX)	From 68 to 100% in urine; preliminary. Sensitivity estimates of 85.9% (95% CI, 72.3–93.4).	94.8% (95% confidence intervals (CI), 92.7–96.4)	The major disadvantage: multiple pipetting processes, extensive incubation time and high cost of antigen. The urine-based latex agglutination test detects mostly active patients and quickly turns negative after a successful response.	[32,39]
Conventional PCR	From 70 to 100%, it depends on the genomic region used and the concentration of DNA in the sample.	100%	Precise results, high specificity and sensitivity. Uncomplicated.	[5,40]
Real-time PCR	Sensitivity 95.6% and 100% in dog (bone marrow samples) and human (peripheral blood and bone marrow) samples.	100%	High performance; lack of standardization; various protocols can be performed.	[41,42]
Immunochromatographic tests (IC)	From 83.4 to 95.8%	90–100%	No need for specialized devices. Small quantities of patient blood samples (from the fingertip).	[33,43,44,45]
ELISA test	From 65.3% and 97.1%, depending on the antigen used.	54.1–99.9%	A sensitive serodiagnosis method of VL that depends on the antigen used.	[46,47,48]
Indirect immunofluorescence assay (IFA)	80–100%	90–100%	Main limitations: Cross-reaction with other trypanosomatids and the low sensitivity in detecting asymptomatic patients.	[45,46,48]

## Data Availability

Not applicable.
